# Rare Intraoperative Presentation of a Coexistent Uterine Cystic Mass and Bilateral Ovarian Cystic Masses

**DOI:** 10.7759/cureus.101386

**Published:** 2026-01-12

**Authors:** Satjeet K Deol Chauhan, Brittany Mckinley, Swati Kumari, Sebastian Reyes Lizaola, Alireza Mehdizadeh

**Affiliations:** 1 Obstetrics and Gynecology, BronxCare Health System, New York City, USA

**Keywords:** benign uterine cysts, coexistent uterine and ovarian cyst, müllerian tumor, uterine cystadenoma, uterine cystic mass

## Abstract

Uterine cystic lesions are commonly encountered in clinical practice, but serosal cysts, such as uterine serous cystadenomas, are exceedingly rare. This case report highlights the rare occurrence of a large uterine serous cystadenoma, initially misdiagnosed as an ovarian cyst on preoperative imaging and later discovered intraoperatively to be an exophytic cystic mass arising from the uterus. It underscores the diagnostic challenge of differentiating large exophytic uterine cystic masses from ovarian cystic masses in patients with distorted anatomy on preoperative imaging. A 50-year-old postmenopausal woman, with no significant past medical or surgical history, was referred for incidental bilateral ovarian cysts detected on CT imaging due to abdominal pain. Transvaginal ultrasound confirmed the cysts, and tumor markers were within normal limits. Given her postmenopausal status, a bilateral salpingo-oophorectomy was planned. During surgery, a 15 cm cyst was found arising from the uterine fundus instead, along with multiple simple cysts on the uterus and small bilateral ovarian cysts. The patient underwent cyst aspiration and excision of the uterine cyst, along with bilateral salpingo-oophorectomy. A hysterectomy was considered due to potential malignancy risk, but was deferred after benign findings on frozen section. Final pathology confirmed a benign Müllerian cyst consistent with serous cystadenoma, with no evidence of malignancy. This case illustrates the importance of intraoperative frozen section analysis and highlights the need for thorough preoperative imaging to guide surgical decisions.

## Introduction

Uterine cystic lesions are a diverse group of pathological entities, and while their occurrence is relatively common, those originating from the serosal surface of the uterus are extremely rare. The majority of uterine cysts are classified as intramyometrial, and serous cystadenomas are typically considered to arise from the ovaries or fallopian tubes. However, cysts originating from the serosal surface of the uterus remain an under-recognized entity in the literature, with limited case reports detailing their clinical presentation and management. These rare tumors are thought to arise from mesothelial cells in the serosal layer of the uterus, potentially originating from remnants of the Müllerian ducts or even from endometriosis. The differential diagnosis often includes more common gynecologic lesions such as fibroids, cystic adenomas, and paratubal cysts [1].

Recent studies have highlighted the role of imaging techniques such as transvaginal ultrasound and CT in the initial identification of such lesions, while intraoperative findings and frozen section analysis are crucial for determining the nature of these cysts and the extent of their involvement. A prior study reported on the coexistence of uterine lipoleiomyoma and bilateral granulosa cell tumors in a postmenopausal woman highlighting the possibility of synchronous uterine and ovarian pathology in this age group [2]. Additionally, the rare occurrence of these lesions has made it difficult to establish standardized guidelines for surgical intervention, with some studies advocating for more conservative approaches based on the benign nature of the cysts [3].

## Case presentation

**Figure 1:**
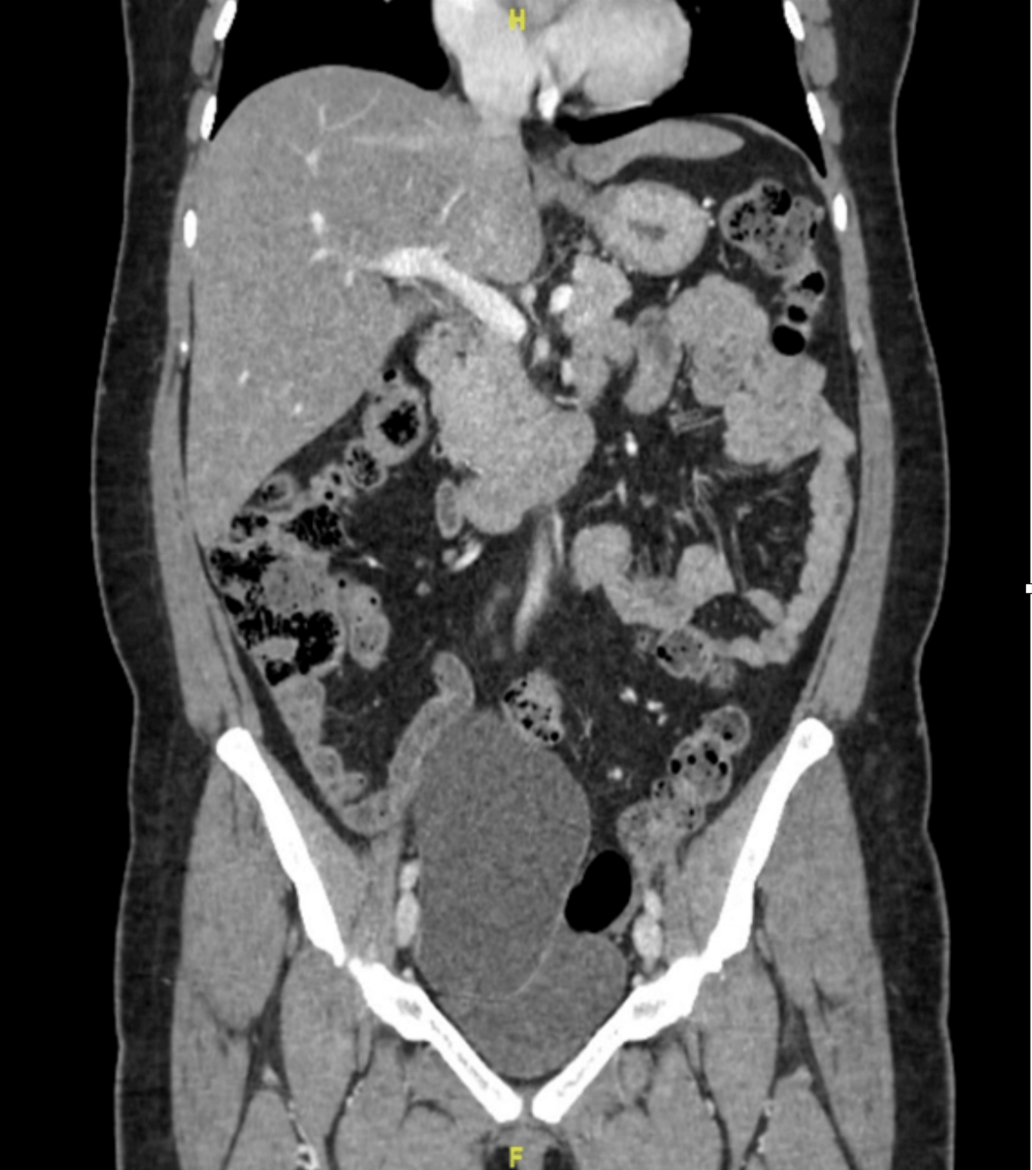
CT imaging showing a left sided adnexal cyst

**Figure 2:**
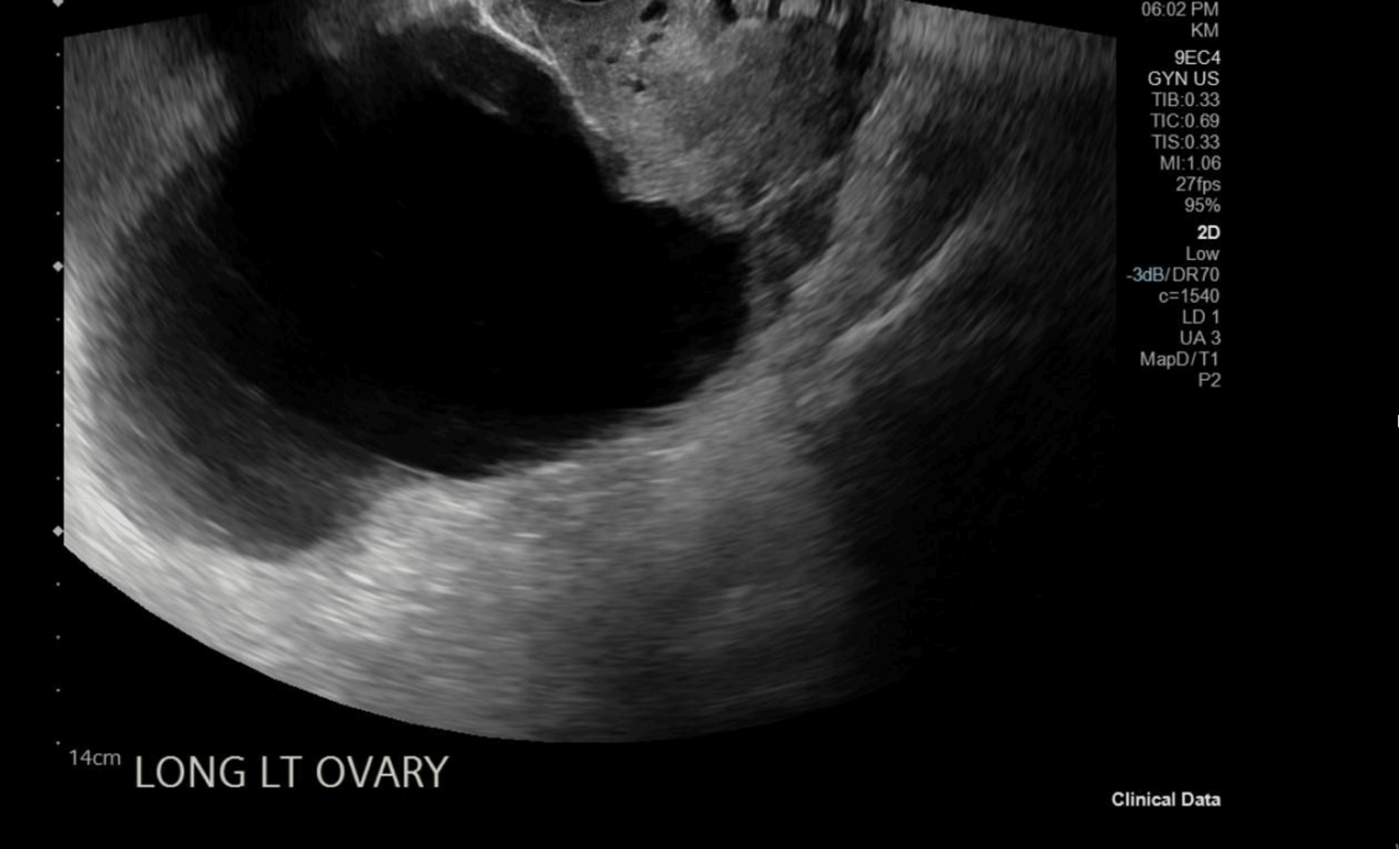
Transvaginal ultrasound imaging showing the left ovarian cyst

A 50-year-old postmenopausal woman, gravida 0 para 0, with no significant medical or surgical history, was referred for the incidental finding of bilateral ovarian cysts on CT imaging done for evaluation of abdominal pain (Figure 1). She underwent a transvaginal ultrasound for better characterization of the cystic masses and Doppler evaluation. The left ovarian cyst measured 13.6 x 9.5 x 10.6 cm, and the right ovarian cyst measured 3.8 x 3.1 x 3.9 cm (Figure 2). The tumor markers were tested, and all were within normal limits. Given the postmenopausal status and bilateral simple ovarian cysts, the decision was made to proceed with bilateral salpingo-oophorectomy.

**Figure 3:**
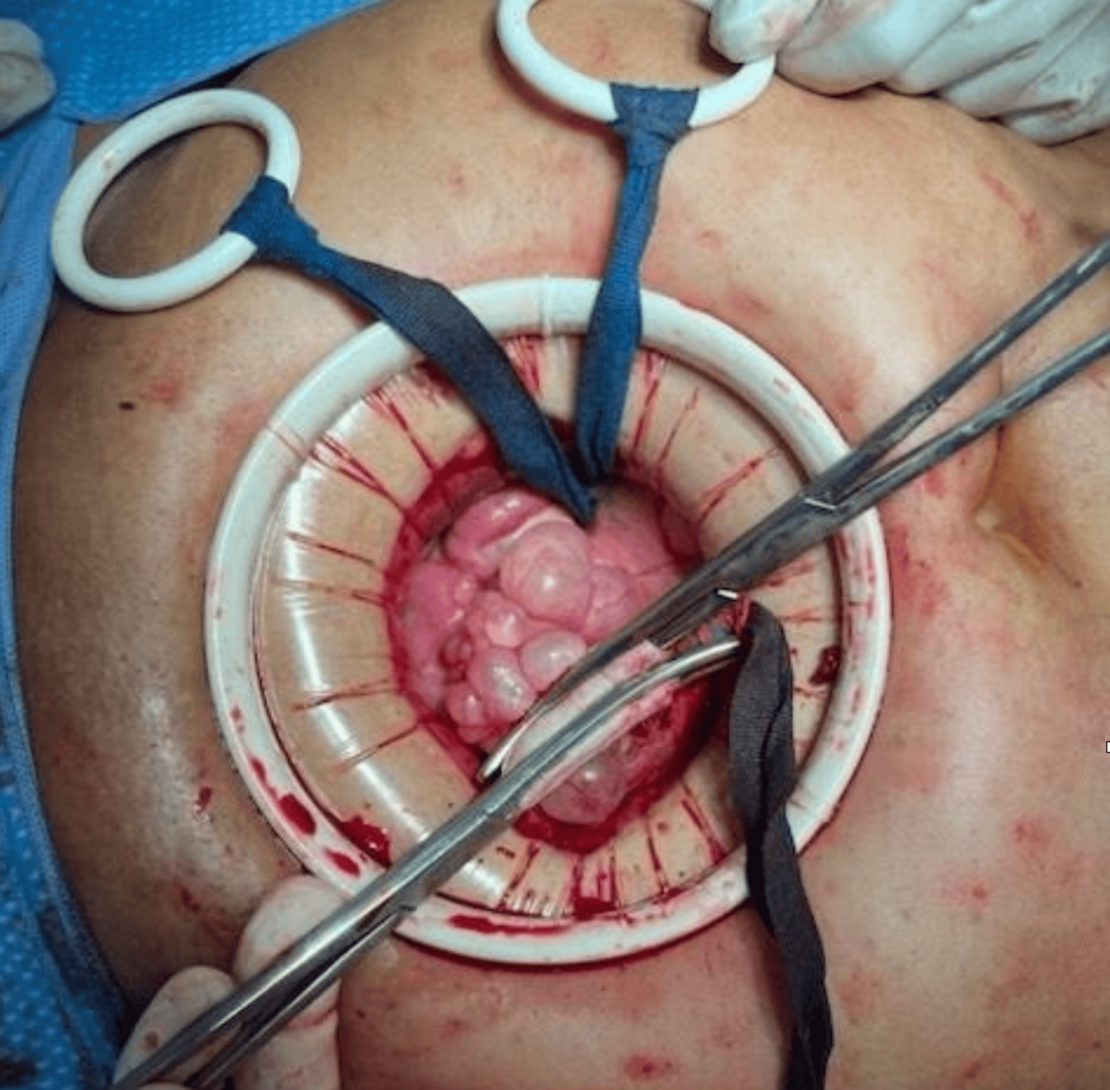
Intraoperative image showing the pedicle of a removed large uterine cystic mass with multiple small uterine cysts.

Intraoperatively, a 15 cm cyst was seen arising from the uterine fundus instead of the ovary, along with numerous simple appearing cysts on the fundal and posterior aspects of the uterus containing clear fluid (Figure 3). Bilateral ovarian cysts, along with multiple paratubal cysts, were also seen at the proximal portion of the bilateral fallopian tubes. She underwent exploratory laparotomy with cyst aspiration by the Pelosi method (controlled cyst decompression to minimize spillage and facilitate removal), along with uterine cyst excision and bilateral salpingo-oophorectomy for small bilateral ovarian cysts.

**Figure 4:**
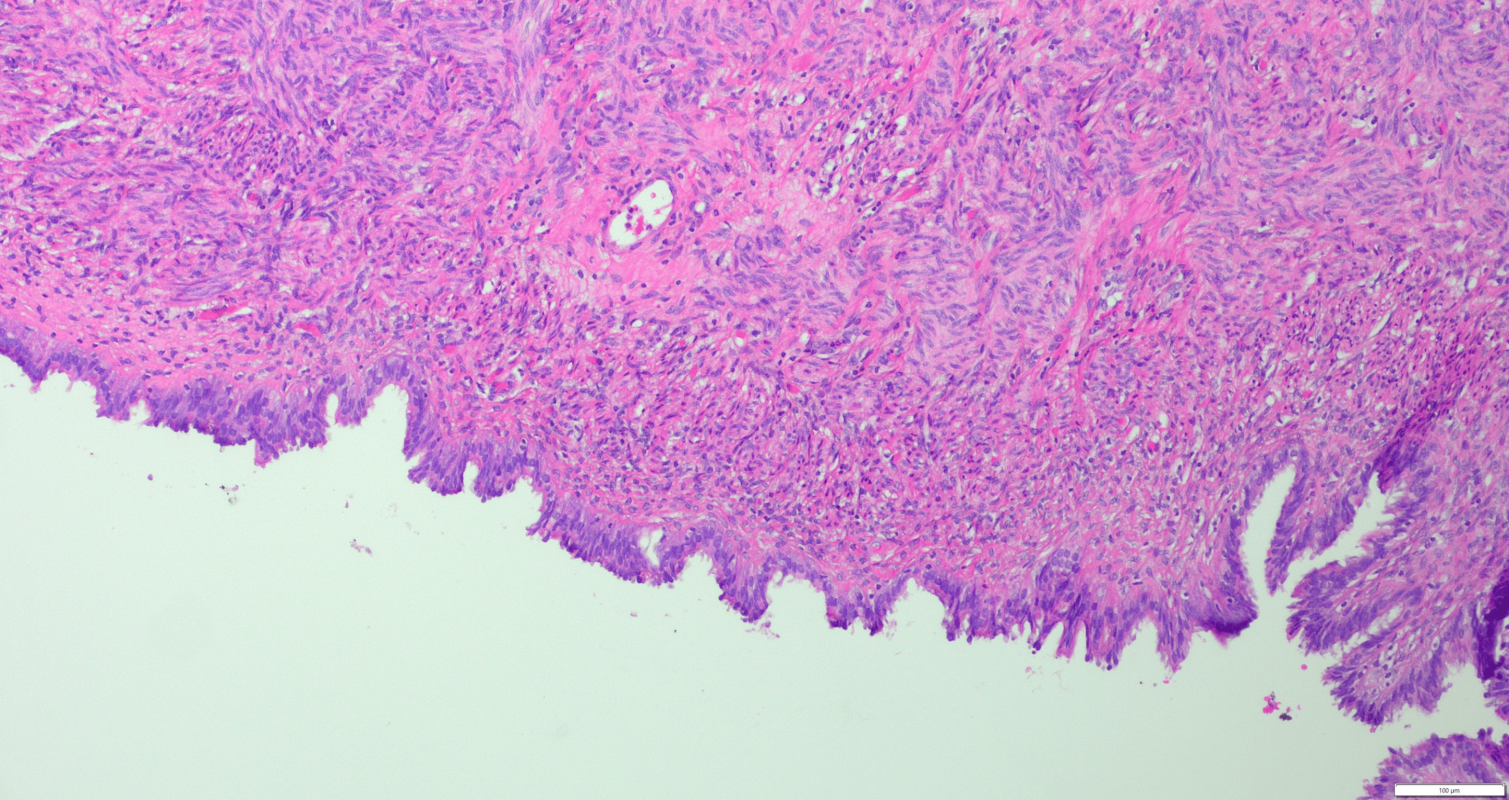
A cyst lined with Müllerian epithelium

Due to the finding of numerous surface epithelial cysts and paratubal cysts and the potential for malignancy/ cyst recurrence, a hysterectomy was considered. However, since the patient had not consented to a hysterectomy, a frozen section was requested from the cyst wall, which was reported as a benign Müllerian cyst. Given the benign findings on frozen section and her consent was limited to bilateral salgingo-oophrectomy, hysterectomy was deferred. Postoperatively, the patient was informed about the intraoperative findings and the option of later surgery to remove her uterus; however, the patient chose to follow up with frequent surveillance. Her final pathology was consistent with a benign Müllerian cyst, consistent with serous cystadenoma, with no histologic evidence of borderline features or malignancy noted (Figure 4).

## Discussion

Rarity and clinical significance

The unusual aspect of this case lies in the coexistence of cystic lesions in multiple pelvic sites. Most uterine cysts are secondary to degenerative changes in leiomyomas or arise from congenital remnants, while cystadenomas are typically ovarian in origin [4]. Encountering a uterine serous cystadenoma in conjunction with bilateral ovarian cysts broadens the clinical perspective. The synchronous appearance suggests a possible shared embryological basis, likely related to Müllerian duct derivatives, or a common hormonal or developmental trigger. Previous literature has proposed that remnants of paramesonephric ducts may persist and undergo cystic change, providing a plausible explanation for such co-occurrence [5].

From a clinical standpoint, these findings are not trivial. In postmenopausal patients, adnexal cystic lesions always raise concern for malignancy. The additional presence of a large uterine mass heightens this concern. Thus, any intraoperative finding of multiple cystic lesions necessitates a systematic approach to diagnosis and management, guided by frozen section analysis and oncologic principles.

Role of preoperative imaging

Preoperative imaging plays a pivotal role in determining the origin and characteristics of pelvic cystic lesions. In this case, the bilateral ovarian cysts were initially identified on CT and confirmed on transvaginal ultrasound, with the left ovarian cyst measuring over 13 cm. However, the uterine cystic mass was only appreciated intraoperatively, underscoring the limitations of CT and transvaginal ultrasound in delineating extra-ovarian cystic lesions [6]. Magnetic resonance imaging (MRI) is superior in characterizing uterine versus ovarian masses, and its use may have better predicted the presence of a uterine serosal cystadenoma in this patient [7]. MRI provides better tissue delineation than CT and is more accurate in identifying the exact tissue origin in the case of large pelvic masses. This case, therefore, underscores the importance of comprehensive preoperative imaging in guiding surgical planning.

Intraoperative management and ethical considerations

During surgery, the team encountered a 15 cm cyst arising from the uterine fundus, multiple smaller surface cysts, paratubal cysts, and bilateral ovarian cysts. Given the patient’s postmenopausal status, the risk of malignancy was appropriately considered. The decision-making process hinged on an intraoperative frozen section, which revealed a benign Müllerian cyst consistent with a serous cystadenoma. This result allowed for a conservative approach, with excision of the cyst and bilateral salpingo-oophorectomy while avoiding unnecessary hysterectomy.

A central ethical element was patient consent. The patient had consented only to bilateral salpingo-oophorectomy, not hysterectomy. Even though intraoperative suspicion might have justified a more extensive procedure, the surgical team appropriately deferred hysterectomy after confirmation of benign pathology, respecting the patient’s autonomy [8]. This case underscores the importance of detailed preoperative counseling. Informed consent should cover not only the planned procedures but also potential intraoperative contingencies, including the possibility of hysterectomy if malignancy is suspected.

Conservative versus radical surgery

The conservative approach in this case aligns with existing recommendations for uterine preservation when pathology confirms benign lesions. Avoiding hysterectomy minimized operative morbidity while ensuring oncologic safety. Over-treatment, especially in benign gynecologic conditions, can significantly impact patient quality of life and should be avoided. This case, therefore, illustrates a broader principle in gynecologic surgery: tailoring management to the individual, balancing the need for safety with the principle of surgical restraint [9].

Pathogenesis and research implications

The coexistence of uterine, ovarian, and paratubal cysts invites questions about their underlying pathogenesis. Several theories exist. One suggests that embryological remnants of the Müllerian ducts may persist and undergo cystic change. Another proposes metaplastic transformation of mesothelial tissue lining the peritoneum or uterine serosa. Hormonal influences may also contribute to the development of synchronous cysts [10]. Future research, particularly genetic and molecular studies, may clarify whether these lesions share a common developmental pathway or arise independently. Understanding these mechanisms has implications not only for diagnosis but also for counseling patients regarding recurrence risk and long-term outcomes.

## Conclusions

This rare intraoperative presentation of a uterine serous cystadenoma with bilateral ovarian and paratubal cysts underscores several key lessons. First, thorough preoperative imaging, particularly with MRI, should be prioritized to better delineate the origin of pelvic cystic lesions. Second, the intraoperative frozen section remains a cornerstone in guiding surgical decisions, especially in postmenopausal patients where malignancy risk is higher. Third, detailed informed consent is essential to prepare patients for possible intraoperative contingencies. Finally, the case highlights the need for continued research into the embryological and molecular underpinnings of synchronous cystic lesions in the female pelvis. Ultimately, individualized, patient-centered care remains the cornerstone of safe and ethical gynecologic practice.
